# Author Correction: Microstructure of a heavily irradiated metal exposed to a spectrum of atomic recoils

**DOI:** 10.1038/s41598-023-36723-y

**Published:** 2023-06-15

**Authors:** Max Boleininger, Daniel R. Mason, Andrea E. Sand, Sergei L. Dudarev

**Affiliations:** 1 UK Atomic Energy Authority, Culham Centre for Fusion Energy, Oxfordshire, OX14 3DB UK; 2 Department of Applied Physics, Aalto University, 00076 Aalto, Espoo, Finland

Correction to: *Scientific Reports* 10.1038/s41598-022-27087-w, published online 30 January 2023

The original version of this Article contained errors in the Results and Discussion section, under the subheading ‘Properties of the steady state’,

“The energy required to melt a crystal with initial temperature $E_{\rm melt}^0 = c_{\rm V} (T_{\rm melt} - T_0) + L$ is formally obtained by

$E_{\rm melt}^0 = c_{\rm V} (T_{\rm melt} - T_0)+ L^{51,26},$ where $c_{\rm V} \approx 3 k_{\rm B}$ is molar heat capacity, […]”

now reads:

“The energy required to melt a crystal with initial temperature *T*_0_ is formally obtained by $E_{\rm melt}^0 = c_{\rm V} (T_{\rm melt} - T_0)+ L^{26},$ where $c_{\rm V} \approx 3 k_{\rm B}$ is molar heat capacity, […]”

The original Article has been corrected.

