# Supplementary material for: Microstructure of a heavily irradiated metal exposed to a spectrum of atomic recoils
Source: Sci Rep. 2023 Jan 30;13:1684. doi: 10.1038/s41598-022-27087-w (PMC9886950; doi:10.1038/s41598-022-27087-w)
Supplement: Supplementary file 1 — Supplementary Information. [file 41598_2022_27087_MOESM1_ESM.pdf]

☙ Supplemental Material ❧

Microstructure of a heavily irradiated metal exposed to a spectrum of atomic recoils

Max Boleininger,[1,*] Daniel R. Mason,[1,†] Andrea E. Sand,[2,‡] and Sergei L. Dudarev[1,§]

[1] *UK Atomic Energy Authority, Culham Centre for Fusion Energy, Oxfordshire OX14 3DB, United Kingdom*

[2] *Department of Applied Physics, Aalto University, 02150 Espoo, Finland*

(Dated: January 18, 2023)

S1. ELECTRON-PHONON DAMPING

For atoms with temperature below their melting temperature, a damping term is added to model energy loss due to electron-phonon coupling. Following Eqs. (8) and (10) by Mason[1], the electron-phonon damping constant is given by

$$\bar{B}_0 = \frac{\pi m \hbar}{3} \lambda \left\langle \omega^2 \right\rangle D(\epsilon_{\mathrm{F}}), \tag{S1}$$

where m is the atomic mass, $\hbar$ is the reduced Planck constant, λ is the unit-less first reciprocal moment of the Eliashberg spectral function[2], $\langle \omega^2 \rangle$ is the second moment of the phonon density of states, and $D(\epsilon_{\mathrm{F}})$ is the electron density of states at the Fermi level. The electron-phonon stopping force on atom i is then given by

$$\boldsymbol{f}_{\mathrm{e-ph},i} = -\bar{B}_0 \boldsymbol{v}_i, \tag{S2}$$

where $\boldsymbol{v}_i$ is the velocity of atom i. This damping force can be implemented in LAMMPS using various commands, such as FIX ELECTRON/STOPPING, FIX LANGEVIN for absolute zero temperature, or FIX VISCOUS. We used FIX ELECTRON/STOPPING as it allows specifying at which kinetic energy a given damping applies. The effective electron-phonon time-damping constant is obtained by the relation

$$\tau_{\mathrm{e-ph}} = \frac{m}{\bar{B}_0}. \tag{S3}$$

The damping constants for the materials considered in this study are listed in table S1.

S2. ELECTRONIC STOPPING CONSTANT

The electronic stopping force on an atom i is given similarly by

$$\boldsymbol{f}_{\mathrm{ele},i} = -\tilde{B}(v) \boldsymbol{v}_i, \tag{S4}$$

from which the effective electronic stopping time-damping parameter is obtained by the relation

$$\tau_{\mathrm{ele}}(v) = \frac{m}{\tilde{B}(v)}, \tag{S5}$$

TABLE S1. Electron-phonon damping constants.

material	λ	$\hbar^2\langle\omega^2\rangle$ (meV^2)	$D(\epsilon_\mathrm{F})$ (eV^{-1})	$\bar{B}_0$ (eV fs Å^{-2})	$\tau_\mathrm{e-ph}$ (ps)
bcc-W	0.26[4]	425[4]	0.355[1]	1.188	16.0
fcc-Cu	0.16[5]	496[5]	0.30[6]	0.249	26.4
hcp-Zr	0.45[7]	249[8]	0.45[7]	0.757	12.5

where $\tilde{B}(v)$ is the velocity-dependent electronic damping. This damping is only applied to atoms with a kinetic energy above 10 eV using the FIX ELECTRON/STOPPING command in LAMMPS. The electronic damping is related to the electronic stopping power $S(E)$ by the expression

$$\tilde{B}(v) = S(E)/v. \tag{S6}$$

The electronic stopping power as a function of ion kinetic energy is obtained from the "Ion Stopping and Range Tables" of the SRIM code[3]. At low ion velocities, corresponding to kinetic energies of below $\sim$100 keV for the materials considered here, we found the time-damping parameter to be approximately constant with respect to velocity, and as such it is well approximated by the low velocity limit. We note that it is also possible to directly import the entire stopping range table into LAMMPS if needed.

S3. CASCADE MODEL PARAMETERS

First, the average and minimum threshold displacement energy E_d and $E_\mathrm{d}^\mathrm{min}$, respectively, are determined for the EAM potentials used in this study. We used a method broadly following Nordlund *et al.*[9].

Molecular dynamics simulations were ran with initial temperature at absolute zero, using the electronic stopping and electron-phonon damping models defined previously. We applied a recoil energy E_R to a randomly chosen atom with a randomly oriented velocity, evolved the system for 5 ps, and tested if at least one defect was produced using the Wigner-Seitz analysis method. This process was repeated many times over a range of recoil energies between 5 eV and 300 eV, depending on the material, recording for each simulation the recoil energy, recoil unit velocity, and number of generated defects. Recoil energies were converted into damage energies using the Lindhard stopping formula. Using the recorded data, $E_\mathrm{d}^\mathrm{min}$ was defined as the lowest damage energy at which a recoil successfully generated a defect. To obtain E_d, we filtered the data for successful events, and multiplied the unit velocity of each data point by the transformation matrices of the symmetry groups of the crystal lattice (bcc and fcc: O_h, hcp: $\mathrm{D}_{6\mathrm{h}}$). The resulting unit vectors were assigned to their nearest vertices on a geodesic sphere (6 frequency subdivision). The minimum damage energy was determined for each vertex. The displacement threshold energy E_d was defined as the minimum damage energy averaged over the vertices.

The same simulation setup was used for single cascade simulations in order to obtain the number of generated defects, this time with recoil energies up to 100 keV. System sizes were varied between 65,000 and 2,000,000 atoms, with simulations repeated until the mean error of the number of generated defects for a given recoil energy reached below 5 %. Simulations for determining the number of heat-spike atoms were done in the same setup, except that they were terminated after 1 ps, at which point the heat-spike has began subsiding. For

TABLE S2. Heat-spike parameters, $x = (c/a)\sqrt{3/8}$.

crystal	r_{cna} [a] (a)	N_{coord}	r_{coord} [b] (a)
bcc	1.207	6	0.933
fcc	0.8536	8	0.8536
hcp	$(1+\sqrt{(4+2x^2)/3})/2$	8	1.300

[a] This choice follows the recommendation in the LAMMPS manual.
[b] This cutoff radius lies between the first and second neighbours.

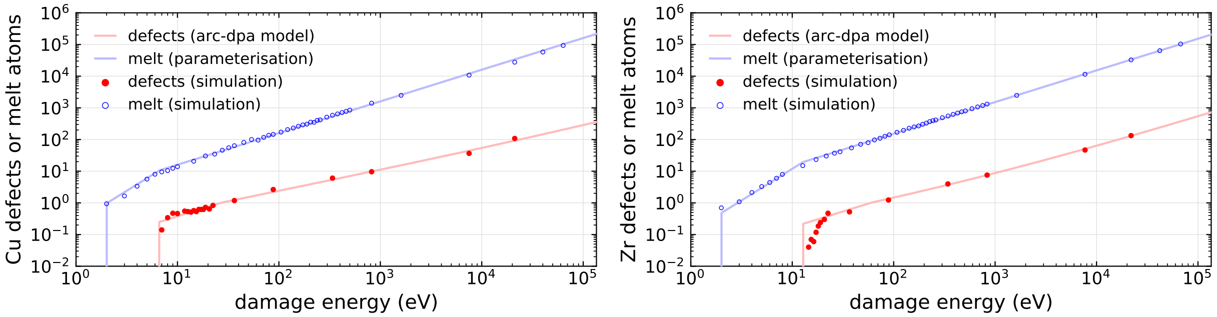

FIG. S1. Simulation and model for the mean number of defects and molten atoms generated by a cascade with given damage energy in copper and zirconium.

determining the heat-spike atoms, first common-neighbour analysis is applied using the cutoff distance r_{cna}, filtering out atoms that are part of the stable crystal structure. Next, atoms are filtered out with a coordination number below N_{coord}, using the cutoff distance r_{coord}. The remaining atoms are defined to constitute the heat-spike. The common-neighbour and coordination number analysis parameters used for determining the heat-spike atoms are listed in table S2.

Figure S1 shows the defects and molten atoms generated by a cascade in copper and zirconium for the interatomic potentials used here. Parameters for the arc-dpa and melt models obtained by fitting the curves are listed in table S3.

S4. DISSOLUTION OF A VOID BY LOW RECOIL ENERGY CASCADES

A void of 3 nm diameter, containing 1061 vacancies, is created by deleting a sphere of atoms in an initially pristine bcc tungsten crystal with dimensions of $32 \times 32 \times 32$ unit cells. Periodic boundary conditions are used. The system is subjected to collision cascades with a recoil energy of 100 eV while the number of vacancies constituting the largest vacancy cluster is monitored, see Fig, S2. It is found that the void dissolves completely over the course of irradiation. This finding suggests that large voids are not inherently stable with respect to low energy irradiation. Curiously, the dissolution process is not driven by interstitial migration, as a small number of interstitials are present only at low dose, but rather by ballistic ejection of vacancies from the void into the surrounding crystal.

TABLE S3. Arc-dpa and melt model parameters obtained from single cascade simulations.

material	$E_{\rm d}^{\rm min}$ (eV)	$E_{\rm d}$ (eV)	b	c	$E_{\rm melt}^{\rm min}$ (eV)	$E_{\rm melt}$ (eV)
bcc-W	46.8	106.0	-0.810	0.227	8.0	2.70
fcc-Cu	6.6	10.6	-0.350	0.021	2.0	0.63
hcp-Zr	12.7	23.5	-0.402	0.283	2.0	0.66

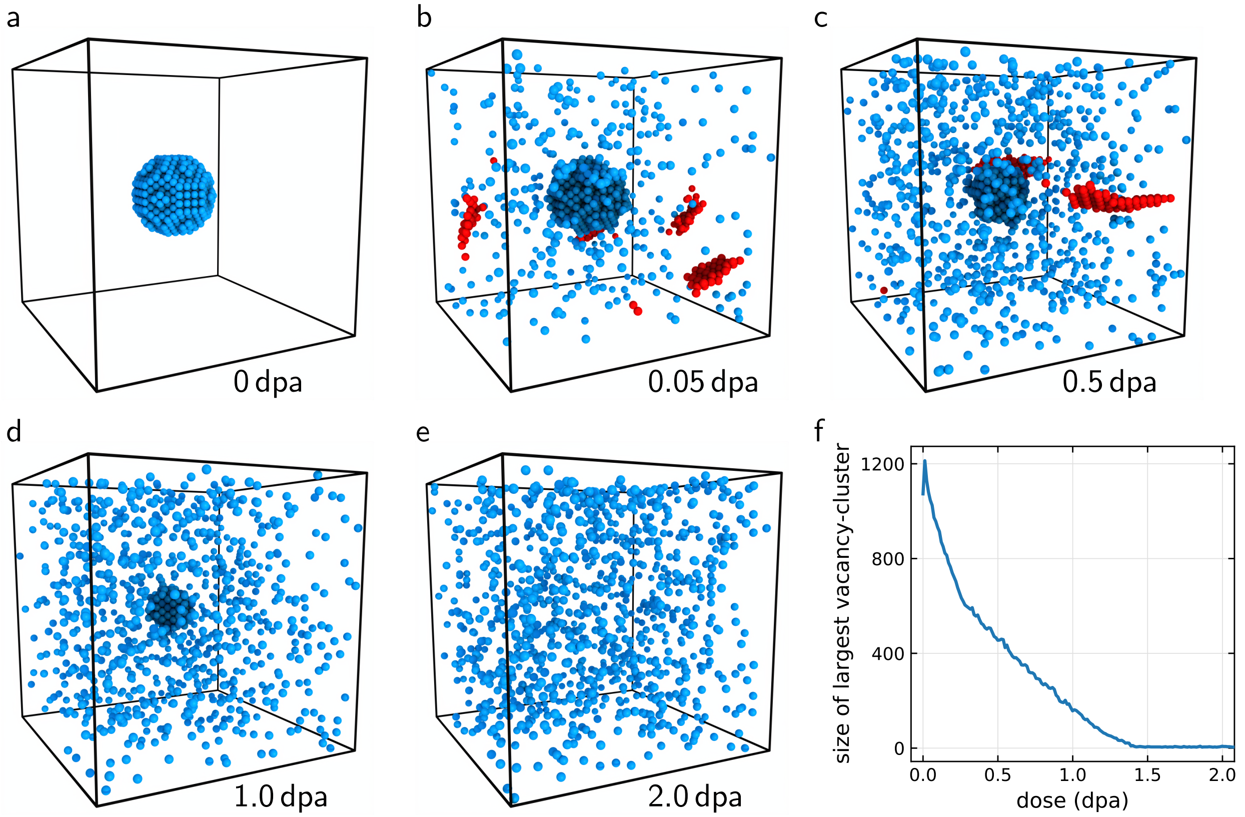

FIG. S2. **a-e** Dissolution of a 3 nm diameter void cluster in tungsten by cascades with 100 eV recoil energy. **f** Size of the largest void cluster in number of vacancies as a function of irradiation dose. The void is completely dissolved after a dose of 1.5 dpa.

S5. PREDICTED DEUTERIUM CONCENTRATION WITH FITTED MODEL

The fitted model shown in Fig. 4b of the article uses the following parameters: $E_{\rm d}^{\rm min} = 47.91\,{\rm eV}$, $E_{\rm d} = 42.52\,{\rm eV}$, $b = -0.6$, $c = 0.23$, with the number of defects of the arc-dpa model further scaled by a factor of 0.43. The melt model uses the parameters shown in table S3. The predicted deuterium concentration using the fitted model is shown in Fig. S3. The fitted model matches the cascade results better at low damage energies than the predicted model, and therefore shows better agreement for light ion irradiation. The recoil spectrum for HFIR neutrons is shown in figure S3.

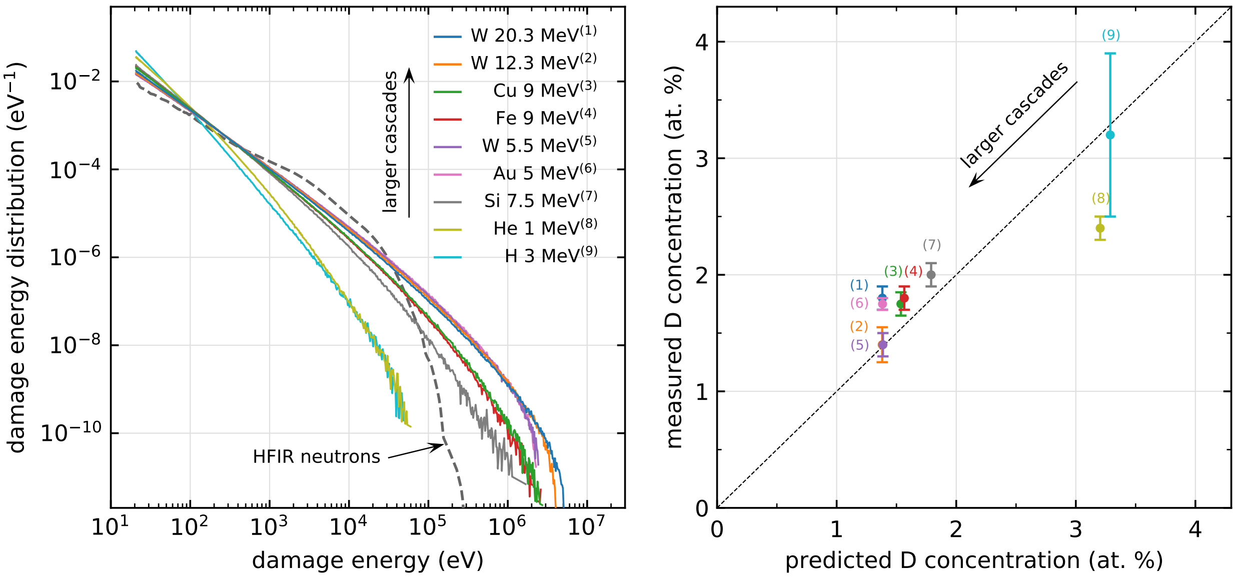

FIG. S3. **a** Distribution of cascade damage energies for various irradiation ions and for irradiation neutrons produced in HFIR. **b** Comparison of analytical and experimentally measured deuterium concentrations in the saturated limit. The analytical deuterium concentration is determined using the fitted vacancy saturation curve shown in Fig. 4b.

[1] D. R. Mason, Incorporating non-adiabatic effects in embedded atom potentials for radiation damage cascade simulations, Journal of Physics: Condensed Matter **27**, 145401 (2015).

[2] Also known as the electron-phonon coupling constant.

[3] J. F. Ziegler, SRIM-2003, Nuclear Instruments and Methods in Physics Research Section B: Beam Interactions with Materials and Atoms **219**, 1027 (2004).

[4] S. Brorson, A. Kazeroonian, J. Moodera, D. Face, T. Cheng, E. Ippen, M. Dresselhaus, and G. Dresselhaus, Femtosecond room-temperature measurement of the electron-phonon coupling constant γ in metallic superconductors, Physical Review Letters **64**, 2172 (1990).

[5] N. A. Lanzillo, J. B. Thomas, B. Watson, M. Washington, and S. K. Nayak, Pressure-enabled phonon engineering in metals, Proceedings of the National Academy of Sciences **111**, 8712 (2014).

[6] O. Bunău and Y. Joly, Self-consistent aspects of x-ray absorption calculations, Journal of Physics: Condensed Matter **21**, 345501 (2009).

[7] W. Butler, Electron-phonon coupling in the transition metals: Electronic aspects, Physical Review B **15**, 5267 (1977).

[8] J.-P. Crocombette, P. Notargiacomo, and M. Marinica, Effect of the variation of the electronic density of states of zirconium and tungsten on their respective thermal conductivity evolution with temperature, Journal of Physics: Condensed Matter **27**, 165501 (2015).

[9] K. Nordlund, J. Wallenius, and L. Malerba, Molecular dynamics simulations of threshold displacement energies in Fe, Nuclear Instruments and Methods in Physics Research Section B: Beam Interactions with Materials and Atoms **246**, 322 (2006).
